# Advances in Pharmacological Actions and Mechanisms of Flavonoids from Traditional Chinese Medicine in Treating Chronic Obstructive Pulmonary Disease

**DOI:** 10.1155/2020/8871105

**Published:** 2020-12-31

**Authors:** Yang Yang, Xin Jin, Xinyi Jiao, Jinjing Li, Liuyi Liang, Yuanyuan Ma, Rui Liu, Zheng Li

**Affiliations:** ^1^State Key Laboratory of Component-Based Chinese Medicine, College of Pharmaceutical Engineering of Traditional Chinese Medicine, Tianjin University of Traditional Chinese Medicine, Tianjin 301617, China; ^2^Military Medicine Section, Logistics University of Chinese People's Armed Police Force, Tianjin 300309, China

## Abstract

Chronic obstructive pulmonary disease (COPD) is a common respiratory disease with high morbidity and mortality. The conventional therapies remain palliative and have various undesired effects. Flavonoids from traditional Chinese medicine (TCM) have been proved to exert protective effects on COPD. This review aims to illuminate the poly-pharmacological properties of flavonoids in treating COPD based on laboratory evidences and clinical data and points out possible molecular mechanisms. Animal/laboratory studies and randomised clinical trials about administration of flavonoids from TCM for treating COPD from January 2010 to October 2020 were identified and collected, with the following terms: chronic obstructive pulmonary disease or chronic respiratory disease or inflammatory lung disease, and flavonoid or nature product or traditional Chinese medicine. Pharmacokinetic studies and external application treatment were excluded. A total of 15 flavonoid compounds were listed. Flavonoids could inhibit inflammation, oxidative stress, and cellular senescence, restore corticosteroid sensitivity, improve pulmonary histology, and boost pulmonary function through regulating multiple targets and signaling pathways, which manifest that flavonoids are a group of promising natural products for COPD. Nevertheless, most studies remain in the research phase of animal testing, and further clinical applications should be carried out.

## 1. Introduction

Chronic obstructive pulmonary disease (COPD) is a devastating lung disease characterized by incomplete reversible air flow limitations that often develop progressively [1]. In the past few decades, COPD has been rifely accepted as a self-inflicted illness attributed to tobacco smoking. Although smoking is the major identified risk factor for COPD, one-third of worldwide patients are nonsmokers [2]. Furthermore, exposure to smoke from burning of biomass fuel and high levels of air pollution are also major environmental risk factors for COPD in many places around the globe [3]. Therefore, some researchers have suggested that COPD is the end result of lifelong, dynamic, and cumulative gene-environment interactions, which modulate the development, maintenance, and function of the lungs through complex and varied biologic mechanisms [4]. More than 3 million people die from COPD, which causes a major healthcare burden worldwide [5]. In 2014-2015, estimated overall prevalence of COPD was 13.6% among Chinese adults aged 40 years and above, indicating that COPD has become a dominating public health challenge in China [6]. Therefore, it is imperative to clarify the pathogenesis of COPD and take corresponding treatment measures.

COPD is a heterogeneous disease with intricate pathogenesis. There is endless inflammatory infiltration in the airways, alveoli, and microvasculature of COPD patients [5]. Besides, impaired immune regulation plays a role in COPD, since inflammation and environmental damage in lungs expose some epitopes for the autoimmune attack [7]. Cell senescence generally leads to decreased proliferation with preserved metabolic activity, resulting in increased inflammation, reduced cell regeneration, and carcinogenesis [8]. In addition to the factors mentioned earlier, the fetal stage has been involved in the pathogeny of COPD, which might be programmed early in life [7]. Currently, a series of therapeutic strategies have been developed to inhibit or avoid the etiologic factors.

Treatment strategies for COPD include smoking cessation, physical activity, and pharmacotherapy. Thereinto, the mainstay of drug treatment for COPD involves inhaled long-acting *β*2 agonists (LABAs), long-acting muscarinic antagonists (LAMAs), inhaled corticosteroids, oral phosphodiesterase-4 inhibitors, and macrolides [5]. Although bronchodilator therapy (LABA, LAMA, or a combination of both) has been shown to be generally safe, there have been adverse cardiac events in clinical studies [9]. Inhaled corticosteroids are related to a higher risk of pneumonia in patients with severe COPD [10]. Phosphodiesterase-4 inhibitors have several side effects such as diarrhoea and nausea [5]. Therefore, novel strategies are needed for better therapeutic response and reduced side effects.

In recent years, an increasing number of studies have shown that flavonoids from traditional Chinese medicine (TCM) have certain protective effect on COPD [11–13]. Although there have been many research studies on flavonoids in treating COPD, the mechanisms of action of flavonoids are still not totally clear and few securable reviews illustrate the comprehensive and multiple pharmacological effects of flavonoids on COPD. Herein, this review aims to illuminate the pharmacologic actions and underlying mechanisms of TCM-derived flavonoids on COPD, based on experimental evidences as well as clinical data to lay the basis for subsequent research studies and developments of anti-COPD medications.

## 2. Flavonoids in TCM

As the most common secondary metabolites, flavonoids are a large group of polyphenolic compounds and exist widely in plants as free aglycones or glycosides. The biosynthesis of flavonoids proceeds *via* the acetate pathway and the shikimate pathway [14]. Constructing from a 15-carbon skeleton, flavonoids are composed of two benzene rings connected by 3-carbon linking chain and mainly classified into flavones, flavonols, flavanone, flavanonol, flavan, flavanol, isoflavone, and chalcone [14].

**Figure 1:**
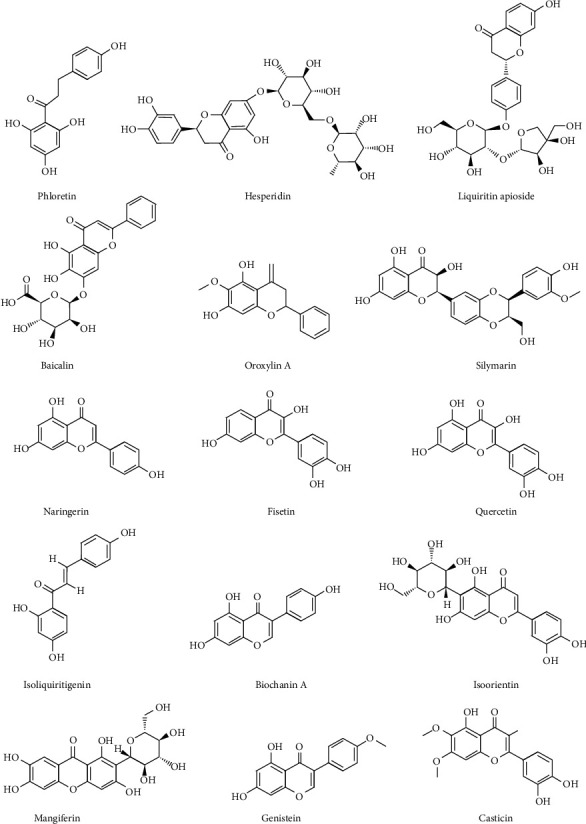
Chemical structure of flavonoids for the treatment of chronic obstructive pulmonary disease.

**Figure 2:**
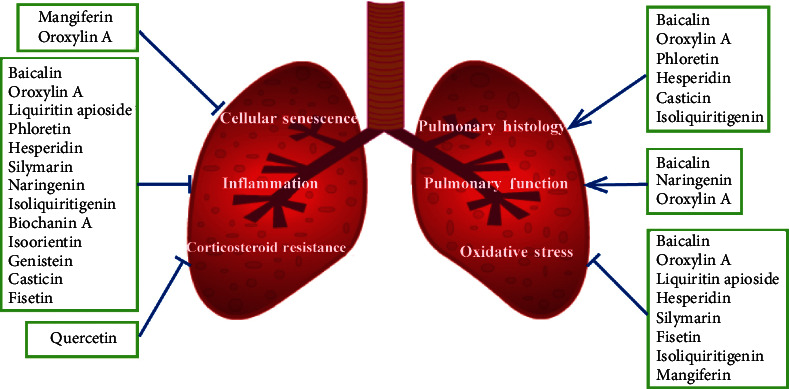
The diverse mechanisms of flavonoids targeting chronic obstructive pulmonary disease, representing the inhibiting effect.

Flavonoids have various functions both in plants and living organisms. The wide variety and high diversity of flavonoids are pivotal for plants to interact with the environment to defense both biotic and abiotic threats [15]. Flavonoids have a variety of biological properties such as antianaphylaxis [16], antibiosis [17], anti-inflammatory [18], vasodilatory [19], antimutagenic [20], and anticarcinogenic activities [21]. Recently, increasing research studies showed the protective effects of flavonoids on COPD through relieving symptoms and improving lung functions [22]. The pharmacological activities and structures of flavonoids for treating COPD are shown in Table 1 and Figure 1, respectively. The schematic diagram of poly-pharmacological properties of flavonoids is displayed in Figure 2.

## 3. Roles of Flavonoids on Chronic Obstructive Pulmonary Disease

### 3.1. Inflammation

COPD is a progressive inflammatory disease of the microvasculature, the alveoli, and the airways, in where inflammation-associated cells including macrophages, neutrophils, eosinophils, and dendritic cells are recruited to form the innate immune response [42]. In COPD, inflammatory tissue damage is endless. Therefore, relieving inflammation has a certain therapeutic effect on COPD.

Isolating from the root of *Scutellariae radix*, baicalin is an isoflavone and has various biological activities. To clarify the effects of baicalin on COPD, the rat model and cell models were established by using cigarette smoke (CS) and cigarette smoke extract (CSE), respectively. The results showed that baicalin reduced production of proinflammatory cytokines and had significant anti-inflammatory effects on COPD [23–25]. The anti-inflammatory effect is likely achieved *via* suppressing the nuclear factor-kappa B (NF-*κ*B) pathway [23] and enhancing histone deacetylase 2 (HDAC2) activity [24].

Oroxylin A, a natural flavonoid isolated from the medicinal herb *Scutellariae radix*, has been proved to have anti-inflammatory and antioxidative properties [43]. Oroxylin A dose-dependently attenuated CS-induced inflammatory cytokine and chemokine production [26].

Liquiritin apioside is a main flavone from *Glycyrrhizae radix et rhizoma*. Liquiritin apioside was reported to attenuate tumor necrosis factor-*α* (TNF-*α*) and transforming growth factor-*β* (TGF-*β*) in human type II alveolar epithelial cell line (A549) [27]. Moreover, liquiritin apioside significantly inhibited goblet cells containing mucus in the atmosphere in a CS-induced mouse model [27].

Phloretin is a chalcone that exists in *Crotonis fructus* and *Rubi fructus* and possesses diverse biologic properties. Phloretin was reported to suppress the mucus hypersecretion and decrease inflammatory in a mice model induced by CS [28]. The results of *in vitro* experiment using NCI–H292 cells were consistent with *in vivo* experiments [28]. The anti-inflammatory effect is achieved *via* restraining phosphorylation of mitogen-activated protein kinase (MAPK) pathways [28]. The above data indicate that phloretin could be as a potential therapy approach to COPD. However, it has not been approved for clinical use.

Hesperidin is a flavone with stable biological activity, which exists in various Chinese herbs such as *Citrus reticulata*, *Schizonepetae herba*, and *Chrysanthemi flos*. *In vivo*, hesperidin was proved to mitigate inflammation in COPD mice by evaluating the levels of related cytokines in bronchoalveolar lavage fluid (BALF) and lung tissues [29]. The mechanism of action of hesperidin was associated with NAD-dependent protein deacetylase sirtuin-1 (SIRT1)/PGC-1*α*/NF-*κ*B signaling axis [29]. These data provide the laboratory evidence of hesperidin in treating COPD patients.

Silymarin is a flavonoid compound extracted from the *Silybl fructus*. Silymarin has been proved to alleviate lung inflammation by regulating extracellular signal-regulated kinase (ERK)/p38 MAPK pathways [30] and suppressing autophagy activation [31]. Silymarin might be an ideal agent treating inflammatory pulmonary diseases, but more clinical research studies are needed.

Naringenin is a plant-derived flavonone with a variety of biological activities. Naringenin was found to significantly decrease inflammatory cells and reduce the levels of proinflammatory cytokines by restraining the NF-*κ*B pathway in the BALF and serum of CS-induced BALB/c mice [32]. However, its role in COPD patients is not yet clear.

**Figure 3:**
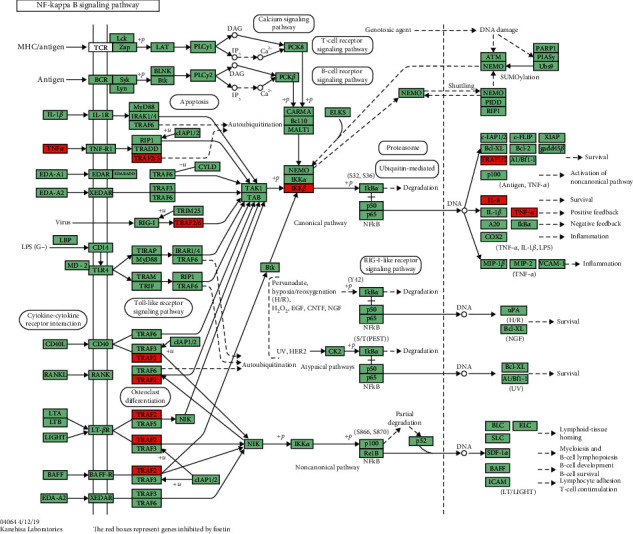
KEGG pathway: map04064.

Fisetin, a plant flavonoid present in *Gleditsiae spina*, has been shown to be effective in lowering inflammation and oxidative stress associated with different disease conditions. Fisetin (25 or 50 mg) demonstrated a significant decrease in inflammatory mediators such as TNF-*α*, granulocyte-macrophage colony-stimulating factor (GM-CSF), and interleukin (IL)-1*β*, IL-4, and IL-10 [44]. The anti-inflammatory mechanism of fisetin was related to suppressing the TNF-*α*-activated NF-*κ*B cascade *via* targeting protein kinase C [33]. The involved proteins in NF-*κ*B pathway that inhibited by fisetin are shown in Figure 3 [45].

Isolated from *Viticis fructus*, casticin is a flavone with various pharmacological effects. In a CS-induced murine model, administration of casticin significantly restrained the influx of inflammatory cells (macrophages, neutrophils, and lymphocytes) and reduced proinflammatory cytokines and chemokines in BALF [34]. These data suggest that casticin could be a promising candidate for suppressing lung inflammation in COPD.

Isoliquiritigenin, a natural chalcone derived from *Glycyrrhizae radix et rhizoma*, has various benefits including antioxidant, anti-inflammation, and antiapoptotic properties [35, 46, 47]. It was reported that isoliquiritigenin could attenuate CS-induced inflammatory cytokines levels (TNF-*α*, IL-1*β*) and the number of cells (neutrophils, and macrophages) in BALF *via* suppressing the NF-*κ*B signaling pathway [48].

Biochanin A is a phytoestrogenic isoflavone of *Triflolium pratense*. It was clearly showed that biochanin A could inhibit the number of total inflammatory cells, neutrophils, eosinophils, and levels of cytokines, including IL-2, IL-4, IL-5, and TNF-*α*, in BALF of the mice [36]. In a PM_2.5_-exposed rat model, Biochanin A decreased apoptosis and the levels of proinflammatory factors including TNF-*α*, IL-2, IL-6, and IL-8 [37].

Isoorientin, vitexin, and isovitexin are natural flavones isolated from *Anthopterus wardii*. Human SAE cells were treated with 5% CSE to evaluate the effects of isoorientin, vitexin, and isovitexin on COPD. The results revealed that these flavones could significantly inhibit the level of IL-8 and MMP-1 at 100 *μ*g/ml [49]. Therefore, isoorientin, vitexin, and isovitexin could play a therapeutic role in the treatment of COPD, but more *in vivo* and clinical research studies are needed to clarify their efficacy and safety.

From the literature mentioned above, flavonoids from TCM could inhibit inflammatory response both *in vitro* and *in vivo* mainly *via* suppressing TNF-*α*/NF-*κ*B and MAPK signaling pathways.

### 3.2. Oxidative Stress

Oxidative stress refers to the overgeneration oxides in the biological system when the organism is exposed to a harmful environment and has a significant driving role in the pathogenesis of COPD [50]. Oxidants consist of reactive oxygen species (ROS) and reactive nitrogen species (RNS) and result from CS and inflammatory cells [51]. Nuclear factor erythroid 2-related factor 2 (Nrf2) is a transcription factor that binds to antioxidant response element and originates the transcription of antioxidant genes in response to oxidative stress [34]. Hence, therapy directed towards increasing Nrf2-regulated antioxidants could be a therapeutic strategy for relieving the effects of oxidative stress in COPD.

Baicalin (80, 160 mg/kg) could significantly reduce oxidant malondialdehyde (MDA) production, which reflects the degree of damage induced by oxidative stress and increases total antioxidant capacity (T-AOC), superoxide dismutase (SOD), and heme-oxygenase (HO)-1 level in in the CS-induced model of COPD [24]. Oroxylin A suppressed CS-induced oxidative lung injury by inhibiting 8-isoprostane and augmenting glutathione (GSH) level [26]. And the antioxidant mechanism of oroxylin A was related to the activation of the Nrf2 signaling pathway. Liquiritin apioside inhibited myeloperoxidase (MPO) activity and increased SOD activity in a dose-dependent manner [27]. Hesperidin was proved to alleviate oxidative stress through reducing oxidants level and increasing antioxidants level [29].

Mangiferin ameliorated oxidative stress *in vitro* through inhibiting ROS level [38]. High-dose silymarin (50 mg/kg/day) administration prevented CS-induced elevation in MDA levels and decrease in SOD activities *in vivo* [30]. Fisetin significantly enhanced lung HO-1, GSH peroxidase-2, reduced GSH, SOD, nitric oxide (NO), and NFR2 levels in CS-exposed rats [33]. Isoliquiritigenin was also reported to attenuate MPO activity and MDA level *via* upregulating the Nrf2 signaling pathway in a dose-dependent manner [48].

### 3.3. Cellular Senescence

Defining as complete and irreversible loss of the replicative capacity in primary somatic cells, cellular senescence has participated in the pathogenesis of COPD [7, 49]. Senescent cells secrete multiple inflammatory proteins known as the senescence-associated secretory phenotype, leading to low-grade chronic inflammation, which further drives senescence [52]. Senescence influences lung structure and inflammatory cells, fibroblasts, and progenitors, making repair and regeneration insufficient [53].

It is clearly shown that mangiferin produced intense cytoprotective effect in bronchial epithelial cells (BEAS-2B) [38]. Furthermore, mangiferin sped up wound healing process and restored proliferation rate of bronchial epithelium [38]. Such protective effects of mangiferin stimulate further *in vivo* and clinical research. Oroxylin A could protect both epithelial cells and macrophages from damage by CS *via* activating the Nrf2 signaling pathway [26].

### 3.4. Corticosteroid Resistance

In contrast to many other inflammatory diseases, corticosteroids are largely ineffective in patients with COPD [40]. Peripheral blood mononuclear cells (PBMCs) from patients with COPD show corticosteroid insensitivity and oxidative stress reduces corticosteroid sensitivity *in vitro* [54, 55]. Corticosteroid insensitivity in COPD lungs explains why high doses of inhaled corticosteroids fail to slow disease progression or reduce mortality [56]. Therefore, increasing sensitivity of corticosteroids may be an effective therapeutic measure for COPD.

Quercetin is a naturally occurring flavone isolated from *Polygoni avicularis herba.* The *in vitro* model, quercetin (10 *μ*M), was competent to restore CSE-induced corticosteroid insensitivity and activate adenosine monophosphate-activated protein kinase (AMPK) pathways in the human monocytic cell line U937 and peripheral blood mononuclear cells [40]. Therefore, combining with corticosteroids, quercetin had the potential to be a novel medication for treating COPD.

### 3.5. Pulmonary Histology

Following CS exposure, there are significant changes in the histology of lungs in experimental animals. In CS-exposed groups, the lung slices showed enhanced peribronchial inflammatory cell infiltration, airway epithelial cell hyperplasia, airway epithelium thickening, alveolar space collapse, interstitial edema, and lumen obstruction by mucus and cell debris. Fortunately, these changes can be alleviated by flavonoid treatment.

Baicalin relieved inflammatory cell infiltration and reduced both the mean linear intercepts and the destructive index [23, 25]. Oroxylin A weakened interstitial edema, soakage of inflammatory cells, and thickened alveolar wall [26]. Phloretin abated peribronchial inflammatory cell infiltration, airway epithelial cell hyperplasia, airway epithelium thickening, and lumen obstruction [28]. Hesperidin mitigated the infiltration level of inflammatory cells, alveolar space, and alveolar sacs [29]. Casticin alleviated inflammatory cell infiltration [34]. Similarly, isoliquiritigenin eased inflammatory cell infiltration and intra-alveolar edema [48].

### 3.6. Pulmonary Function

Biochanin A (20 mg/kg, orally) markedly relieved airway resistance (*R*_*L*_), enhanced pause (*P*_enh_), and increased lung dynamic compliance (*C*_dyn_) values induced by methacholine in sensitized and challenged mice [36].

The whole-body plethysmography method was employed to evaluate the protective effect of naringenin on the pulmonary function of CS-exposed mice [32]. The results showed that naringenin significantly improved the parameters of breathing such as peak expiratory flow (PEF), peak inspiratory flow (PIF), and minute ventilation (MV).

Baicalin was reported to increase the peak expiratory flow rate (PEFR), maximum ventilatory volume (MVV), and the forced expiratory volume in 0.3 s (FEV_0.1_)/functional residual capacity (FRC) in CS-induced rat models [42]. Moreover, baicalin could significantly enhance the ventilatory parameters, including PEF, PIF, and MV in CS-exposed mice [25]. Compared with the model group, CS-induced rats treated with different concentrations of baicalin displayed an increased FEV_0.1_/FRC ratio and vital capacity [24]. Moreover, administration of 40 mg/kg baicalin markedly prevented PaCO_2_ augmentation and increased PaO_2_ level [24]. These results indicated that baicalin exerts a lung function protection role in cigarette smoke COPD rats.

## 4. Roles of Flavonoids in Clinical Testing

Genistein is an isoflavone that exists in various Chinese herbs such as *Iridis tectori rhizoma*, *Sojae Semen Praeparatum*, and *Sojae Semen Nigrum*. Patients with COPD (*n* = 34; age, 71.8 ± 9.0 years) were recruited to assess the effects of genistein on COPD [41]. In the supernatant of lymphocytes in COPD patients, the percentage of NF-*κ*B-positive cells and the concentrations of TNF-*α* and MMP-9 were markedly increased. Compared with control treatment, the levels of NF-*κ*B-positive cells, TNF-*α*, and MMP-9 were reduced by genistein treatment. This clinical research indicated that genistein may provide a strategy for protecting patients with COPD.

Quercetin could inhibit acetylcholine chloride-induced continuous contractions in human bronchial airway smooth muscle strips, which indicated that quercetin could be developed as a bronchodilator [39]. Although quercetin has the potential to be a novel medication treating COPD, the safety of quercetin in patients with COPD is still not clear. A randomised clinical trial was performed to assess safety of quercetin [57]. COPD patients with mild-to-severe lung disease with FVE_1_ ranging between >35% and <80% were recruited and administrated with either placebo or quercetin at 500, 1000, or 2000 mg/day in a dose-escalation manner. The results showed that there are no quercetin-related severe adverse events in the patients based on evaluating lung function, blood profile, and COPD assessment test questionnaire. Therefore, quercetin was safely tolerated up to 2000 mg/day. However, the drawbacks of this trial are the small sample size and the short treatment time of quercetin. Hence, the safety of quercetin should be ascertained in a larger cohort of patients for a longer time.

## 5. Current Trends

COPD is an intractable respiratory disease with complicated pathogenic factors. Increasing studies have shown that flavonoids have certain therapeutic effects on COPD. Some of them exert a variety of pharmacological effects. For example, oroxylin A not only inhibits inflammation, oxidative stress, and cell senescence but also restores damaged lung tissue. Baicalin could relieve symptoms of COPD by restraining the levels of inflammation-associated cytokines and oxidative stress, enhancing pulmonary function. The above findings manifest that the protective effects of flavonoids are relevant to a multitarget mode action. Furthermore, many flavonoids could alleviate inflammation response and oxidative stress *via* regulating NF-*κ*B and Nrf2 signaling pathways, respectively. Based on the structure-activity relationship theory, the collective anti-inflammatory and antioxidant properties could be due to structural similarities. Consequently, exploiting the structural similarity may help to develop flavonoid-based novel lead compounds treating COPD.

However, most research studies on the treatment of COPD with flavonoids are still in the laboratory stage and there are few clinical studies on flavonoids. Consequently, the roles and safety of flavonoids on patients with COPD are vague, which greatly limits the application in the treatment of COPD. Based on the current situation, more clinical research studies are required to elucidate the efficacy and safety of flavonoids. In addition, we found that cigarette smoke or cigarette smoke extracts are commonly used in laboratories to trigger off oxidative stress and inflammation to establish COPD models; however, the pathogenesis of COPD is intricate and about one-third of patients worldwide are nonsmokers. Consequently, the current modeling methods cannot adequately reflect the true situation of COPD patients and new methods are extremely needed to comprehensively show the pathological characteristics of COPD.

## 6. Conclusion

Flavonoids are the most diverse phenolic compounds and are broadly available in Chinese herb medicines, and therefore, COPD treatments with such natural compounds can be considered as a kind of promising alternatives. A mass of laboratory evidences have shown the beneficial effects of flavonoids on COPD pathogenesis *via* inhibiting inflammation, oxidative stress, and cellular senescence, restoring corticosteroid sensitivity, improving pulmonary histology, and boosting pulmonary function. Based on the above scenario, it is essential to establish a research platform to comprehensively uncover the interactions between flavonoids and genes in COPD.

## Figures and Tables

**Table 1:** The effects of flavonoids in TCM on chronic obstructive pulmonary disease.

Flavonoids	TCM sources	Models	Effects
Baicalin (C_21_H_18_O_11_)	*Scutellariae radix*	CS-induced rat model	Inhibition of inflammation; prevention of pulmonary function [23]; reducing oxidative stress [24]
		CS-induced mice model	Reduction of inflammation; protection of pulmonary function [25]
		*In vitro* model using CSE-exposed type II pneumocytes	Prevention of inflammation [23]
		CSE-induced human type II alveolar epithelial carcinoma cell line (A549 cells)	Moderation of inflammation response [25]

Oroxylin A (C_16_H_12_O_5_)	*Scutellariae radix*	CS-induced mice model	Alleviation of inflammation and oxidative stress [26]
		CSE induced BEAS-2B bronchial epithelial cells and RAW264.7 cells	Upregulation of Nrf2 expression and total cellular glutathione level [26]

Liquiritin apioside (C_26_H_30_O_13_)	*Glycyrrhizae radix et rhizoma*	CS-induced mice model	Inhibition of inflammation, myeloperoxidase activity, and increased SOD activity [27]
		*In vitro* model using CSE-exposed A549 cells	Attenuation of cytotoxicity, inflammation, and depleted GSH levels [27]

Phloretin (C_15_H_14_O_5_)	*Crotonis fructus*; *Rubi fructus*	CS-induced mice model	Suppression of the mucus hypersecretion and inflammatory cell release [28]
		CSE-induced NCI–H292 cell model	Moderation of inflammatory cytokines and the phosphorylation of MAPK pathways [28]

Hesperidin (C_28_H_34_O_15_)	*Citrus reticulata*	CSE-induced mice model	Inhibition of inflammation and oxidative stress responses [29]

Silymarin (C_25_H_22_O_10_)	*Silybl fructus*	CS-induced mice model	Suppression of inflammation and oxidative stress [30]
			Attenuation of autophagy [31]
	*Silybl fructus*	CSE-induced BEAS-2B cell model	Moderation of inflammatory cytokines in an autophagy- and ERK/p38 MAPK-dependent manner [31]

Naringenin (C_15_H_12_O_5_)	*Menthae herba*	CS-induced mice model	Protecting pulmonary function and decreasing inflammatory cells and cytokines [32]
		*In vitro* model using CSE-exposed A549 cells	Suppression of inflammation [32]

Fisetin (C_15_H_10_O_6_)	*Gleditsiae spina*	CS-induced rat model	Inhibition of inflammation and oxidative stress; prevention of tissue damage [33]

Casticin (C_19_H_18_O_8_)	*Viticis fructus*	CS-induced C57BL/6 mice model	Inhibition of inflammatory cytokines and chemokines [34]

Isoliquiritigenin (C_15_H_12_O_4_)	*Glycyrrhizae radix et rhizoma*	CS-induced mice model	Reduction of the infiltration of inflammatory cells and cytokines; reversion of lung pathological injuries and oxidative stress levels [35]

Biochanin A (C_16_H_12_O_5_)	*Triflolium pratense*	Male Hartley guinea pigs and female	Suppression of inflammation response [36]
		BABL/c mice model	
		PM _2.5_-induced rat model	Amelioration of inflammation and oxidative stress [37]

Isoorientin (C_21_H_20_O_11_)	*Anthopterus wardii*	*In vitro* model using CSE-exposed human SAE cells	Anti-inflammatory activity [37]

Mangiferin (C_19_H_18_O_11_)	*Anemarrhenae rhizoma*	*In vitro* model using PAH-exposed BEAS-2B cells	Ameliorating oxidative stress, speeding up wound healing and restoring proliferation [38]

Quercetin (C_15_H_10_O_7_)	*Polygoni avicularis herba*	ACH-induced mice model	Relieving precontracted airway smooth muscle [39]
		Patients with COPD	Restoring corticosteroid sensitivity [40]

Genistein (C_15_H_10_O_5_)	*Iridis tectori rhizoma*	Patients with COPD	Suppression of the NF-*κ*B, TNF-*α*, and MMP-9-associated pathways [41]
